# Effectiveness of a Treadmill Training Programme in Improving the Postural Balance on Institutionalized Older Adults

**DOI:** 10.1155/2020/4980618

**Published:** 2020-01-30

**Authors:** Natalia Moya Pereira, Marcel Jean Pierre Massè Araya, Marcos Eduardo Scheicher

**Affiliations:** ^1^Postgraduate Program of Human Development and Technologies, Universidade Estadual Paulista (UNESP), Campus de Rio Claro, Rio Claro, SP, Brazil; ^2^Physical Therapist, São Paulo, SP, Brazil; ^3^Department of Physiotherapy and Occupational Therapy, UNESP, Marília, SP, Brazil

## Abstract

**Background:**

Institutionalized older adults have increased gait and balance impairment compared with community-dwelling older adults. The use of the treadmill for the rehabilitation process has been studied in different groups, but not in the institutionalized elderly.

**Objectives:**

The objective of this study was to assess the effects of a treadmill walking workout program on the postural balance of institutionalized older adults.

**Methods:**

Postural balance was assessed by the Berg Balance Scale (BBS), Short Physical Performance Battery (SPPB), gait speed, and Timed Up and Go Test (TUG) on 37 institutionalized older adults (23 in the intervention group and 14 in the control group). Training consisted of a 20-minute treadmill walking workout carried out twice a week for 10 weeks. Measurements were obtained before and after 10 weeks and with 1 month of follow-up for the intervention group. For the control group, the data were obtained before and after the training period.

**Results:**

Significant improvement occurred in all motor function parameters (BBS: *p* < 0.01; gait speed: *p* < 0.01; gait speed: *p* < 0.01; gait speed: *p* < 0.01; gait speed:

**Conclusions:**

The present results permit us to conclude that a treadmill walking program had positive effects on the postural balance of institutionalized older adults.

## 1. Introduction

The aging process causes changes in different aspects of the human gait, with a reduction in performance and modifications in the system of postural control, affecting activities of daily living. Postural imbalance has a negative impact on the life of the elderly and is one of the major problems of this population [1–3]. Changes in gait patterns are observed with aging, with a reduction in step speed and length, increasing the risk of falling and the functional decline, and, consequently, increasing the possibility of institutionalization and hospitalization [1, 2, 4–7].

Institutionalized older adults have fewer opportunities to participate in daily-life activities and tasks in an independent manner, with greater consequent deleterious effects on the physiological losses inherent to aging and with increased gait and balance impairment compared with community-dwelling older adults [8–10]. Physical activities for institutionalized older adults are crucial for the maintenance of their functional independence or for the reduction of their dependence during activities of daily living (ADLs), in addition to increasing self-esteem [11, 12]. Exercise training has the potential to decrease risk of falls, use of potentially harmful drugs (e.g., antipsychotics), and the dependence on ADLs, besides improving malnutrition and pain, mood (particularly depression), sedentary lifestyle (bed and chair rest), and quality of life [13, 14].

The use of a treadmill for the process of rehabilitation, with or without partial weight support, has been studied in different groups such as patients with Parkinson's disease, patients with sequelae of stroke, and older adults after hip fracture, and gait improvement has been observed, including increased step cadence, increased mean speed, and a consequent reduction in the risk of falls [1, 2, 15–18]. In patients with Parkinson's disease, treadmill training promoted benefits for postural balance and the kinematic parameters of gait, with an increase in gait velocity, step width and the amplitude of hip and ankle movement, and a reduction in double support time. In addition, treadmill training resulted in a longer distance covered, improved transfer from the sitting to the standing position, and increased lower limb strength [15]. In patients with stroke sequelae, treadmill training improved the space-time parameters and the motor quality of the gait compared with other physiotherapy techniques. An explanation to this effect is that treadmill training for 30 minutes corresponds to more than 1000 gait cycles, as compared with less than 50 cycles performed during physiotherapy based on the Bobath concept [19, 20]. The hypothesis is that the benefits of treadmill training described above may occur in institutionalized elderly too.

Some studies have used the treadmill as a component of exercise programs on the institutionalized elderly [21–23]. Despite the positive results of treadmill training obtained for certain populations in aspects such as postural balance and gait, there are no literature data on the effects of treadmill training alone in institutionalized elderly. Treadmill training is believed to provide functional independence due to gains in balance and mobility. Therefore, the objective of the present study was to assess the effects of a treadmill walking program on the postural balance and functional mobility of institutionalized older adults.

## 2. Methods

### 2.1. Study Design

This was a two-arm, nonrandomized, and nonblinded study, conducted from July 2016 to October 2017 on elderly subjects of both sexes, aged 60 years or older residing in long-term care facilities for the elderly (LTCFs) in the city of Marília, SP, Brazil. Due to ethical issues [24], randomization was not possible and the exercise intervention was offered to all the subjects. Those who refused to participate in the intervention exercise program were allocated to the control group. The control group was advised to maintain their regular lifestyle habits during the study.

### 2.2. Sample

Subjects were recruited at three long-term care facilities for the elderly (LTCFs) in the city of Marília, SP, Brazil. In the first contact, a researcher rated the patient's eligibility and proposed to participate in the study if the subject met the eligibility criteria. The procedures involved in the evaluations and the intervention program were explained to the subjects, who then signed written informed consent to participate in the study, which was approved by the Research Ethics Committee of Faculty of Philosophy and Sciences, Marília Campus, SP, Brazil (Protocol 1.803.955) and registered with the database of the Brazilian Registry of Clinical Trials (ReBEC) (RBR-7vznbt).

The eligibility criteria were Functional Ambulation Categories (FAC) ≥ 2 [25], absence of cognitive deficit by Montreal Cognitive Assessment (MoCa), with values higher than 26 [26, 27], ability to walk independently 12.4 m to participate in the gait assessment, and absence of physical and/or functional impairment that would limit treadmill walking. Exclusion criteria were the presence of untreated neurological or cardiorespiratory diseases and/or limitation of the ability to walk on the treadmill, incapacitating visual or hearing deficits that would not permit the investigation and the subject's drop-out during any phase of intervention or evaluation. Subsequently, the patient was scheduled to perform the initial evaluation. After the initial evaluation, the patient was assigned to the control or intervention group based on their willingness.

**Figure 1:**
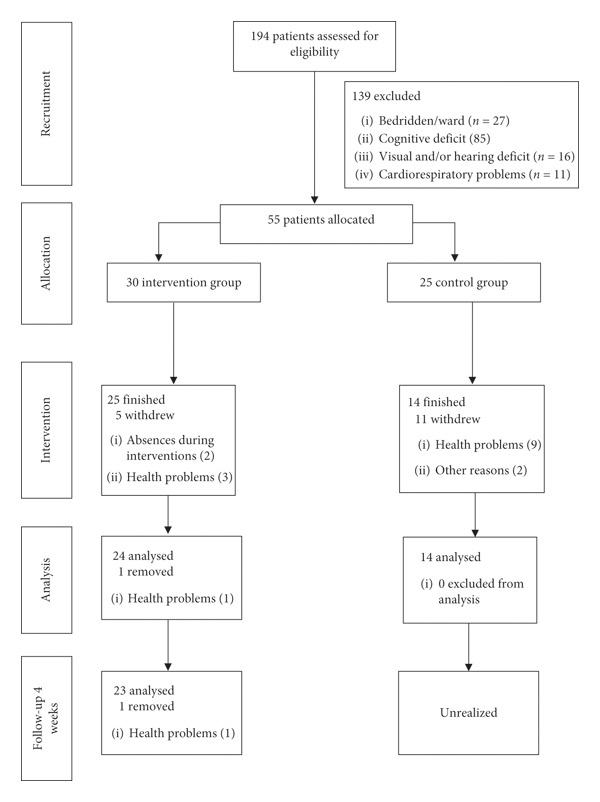
Consort diagram with participant flow.

Figure 1 shows the flow diagram of the study according to inclusion and exclusion criteria, as well as drop-out episodes during evaluation.

### 2.3. Initial Evaluation

Besides the cognitive and FAC evaluation (eligibility criteria), it was applied the 10-meter walk test (10MWT) in order to assess the usual gait speed of the elderly subject, the speed used for familiarization with the treadmill, and the speed used during training. The 10MWT was applied three times in order to reduce the learning effect and to obtain better performance, respectively, conducted before intervention, every 2 weeks during intervention, immediately after intervention, and one month after intervention. This test was used as a parameter variable to evaluate individual evolution every 2 weeks and the increase in speed on the treadmill, as well as for the analysis of training during the 3 phases.

### 2.4. Gait Speed, Mobility, and Postural Balance Evaluation

The gait speed was evaluated using the 10-meter walk test (10MWT), which is safe and easily used with minimal facilities and budget. Before the test, the subjects were warned not to run. Participants were asked to walk with their comfortable gait speed after hearing the Go command, and to eliminate any anomalies, the participants had to begin walking 1.2 meters before the timing and finish 1.2 meters after [7]. During the tests, the examiner did not encourage the volunteers to increase the speed, and using a digital stopwatch with a 1/100 of a second reading (Cronobio SW-2018®, Pastbio, SP, Brazil), the walking time of all volunteers was recorded. The test was carried out three times in order to eliminate any variables, and the shortest durations were used. The use of walking aids was permitted.

The mobility was evaluated by the Timed up and Go (TUG) test. The test measures the time (in seconds) necessary for a person to rise from a chair with armrests, walk 3 meters at a comfortable walking speed, turn, return to the chair, and sit down [28]. The test was performed twice, first for familiarization and then for time recording [28]. The TUG test is highly recommended as a means of assessing the risk of falling for the elderly because it identifies the deficit of balance and gait speed. Therefore, lower scores indicate better functional mobility, better posture, and an increased gait speed [29].

The Berg Balance Scale (BBS) and the Short Physical Performance Battery (SPPB) were used to evaluate the postural balance. BBS was translated, adapted, and validated to Brazil [30] and consists of a battery of 14 tasks common to the ADLs, which quantitatively evaluate the risk of falls, through observation undertaken by the examiner. The SPPB was designed to measure functional status and physical performance, assessing walking speed, standing balance, and sit-to-stand performance and was translated, adapted, and validated to Brazilian Portuguese [31].

### 2.5. Training Protocol

After the initial assessment, the preferred speed for treadmill gait was selected. First, the participant walked on the treadmill for one minute at a speed 50% of that found with the 10MWT until he fully understood the functioning of the equipment. The preferred speed on the treadmill was then calculated and increased until the participant stated that he was walking faster than usual, followed by a reduction until the participant stated that he was walking slower than usual. This process was repeated four times with resting intervals, the mean of the reported speeds was calculated, and the speed of familiarization with the treadmill and the first training periods was defined [32].

The intervention phase consisted of gait training on the treadmill twice a week with intervals of 2 and 3 days during the week, in sessions of up to 40 minutes over a period by 10 consecutive weeks. A treadmill (Movement® Fitness Equipment) was used for the intervention protocol. During the first 2 weeks, the subject walked at his/her familiarization speed, and the mean speed (10MWT) was re-evaluated every 2 weeks, with the possibility of readjustment of speed on the treadmill.

Each session started with 10 minutes of warm-up (stretching mainly of the lower limbs, ten right and left hip rotations, and ten arm rotations forward and backward), followed by treadmill walking for up to 20 minutes and ended with a phase of 10 minutes of cool-down (stretching and relaxation with the elderly in the supine position, resting). During the training, the subject used a safety belt connected to a steel cable fixed to the wall in order to prevent falls, and the investigator corrected the posture during walking with verbal instructions. A pause was granted if the subject required it and/or the session was interrupted.

During the intervention period, data were surveyed on the occasion of each session, such as time of treadmill walking and pauses and distance covered. Blood pressure, heart rate, respiratory rate, and oxygen saturation were evaluated before, during (every 4 minutes), and after each training session as criteria for stopping the training. Each participant was evaluated and trained always at the same time of the day in order to reduce changes in performance related to circadian rhythms.

### 2.6. Statistical Analysis

Data normality was determined using the Shapiro–Wilk test. The data of the intervention group were compared by one-way repeated measures ANCOVA adjusted for age and baseline values as covariates, followed by the Bonferroni post hoc test. The comparison between the intervention and control group was made by ANCOVA adjusted for the baseline values in the respective measurements and for the age, followed by the Bonferroni post hoc test. A significance level ≤0.05 was accepted for all comparisons.

## 3. Results

Table 1 shows the baseline data of the participants, including sex, age, weight, height, number of medications, duration of institutionalization, and cognition. There was no significant difference between groups.

Table 2 shows the comparison of the tests before and after training and with 1 month of follow-up for the intervention group. The differences (calculated by a one-way repeated measures ANCOVA, adjusted for age and baseline values) were SPPB: *F* = 10.98, *p* < 0.0001; BBS: *F* = 38.89, *p* < 0.001; TUG: *F* = 18.64, *p* < 0.0001; and GS: *F* = 47.23, *p* < 0.0001.

Table 3 shows the comparison of the variables analyzed before and immediately after training for the intervention group and the control group. At 10 weeks, the intervention group had greater decreases in TUG values than the control group (−2.26 s vs. 0.26 s (95% CI, 10.6 to 12.2 vs. 14.1 to 16.1); *p* < 0.0001). In addition, the intervention group had greater improvement in BBS (2.64 vs. 0.29 (95% CI, 50.3 to 52.7 vs. 45.2 to 48.2); *p* < 0.0001), SPPB (2.04 vs. −0.68 (95% CI, 9.3 to 11.2 vs. 4.5 to 6.9); *p* < 0.0001), and GS (0.18 vs. 0.07 m/s (95% CI, 0.97 to 1.1 vs. 0.68 to 0.86); *p* < 0.0001) than the control group.

## 4. Discussion

As part of the aging process, older adults experience physical changes in the postural control systems. For various reasons, these changes are more pronounced among institutionalized older adults, increasing the risk of falls. The institutionalized elderly spend most of their time sitting or lying down [33], even if they are able to perform day-to-day tasks, accelerating processes inherent to aging, such as changes in the balance control system. The study of methods aiming at improving the balance of this population is a challenge for investigators in the aging area. The objective of the present study was to assess the effects of a treadmill walking program on the postural balance of institutionalized older adults.

The results revealed significant improvement in all variables of the postural balance not only after training, but also the maintenance of the improved values after one month of follow-up after training, with a significant difference compared with the pretraining period.

The comparison between control and intervention groups also showed a significant difference in the posttraining period. To show that the mean differences or interactive effects are not occurred by chance, the scores on the dependent variables (TUG, EEB, and gait speed) were adjusted for the effect of the covariates (baseline values and age).

Several studies have indicated that a regular practice of specific physical exercises with a high challenge to the balance system can increase gait and postural balance skills of the older adults, reducing the risk of falling and the occurrence of injuries [8, 34–36]. In a systematic review and meta-analysis, Sherrington et al. found no evidence that exercise as a single intervention can prevent falls in long-term care facilities for the elderly [37]. It should be noted that our study did not evaluate the number of falls before and after training. Therefore, it is not possible to say that treadmill training decreased the number of falls. But, based on the reference scores of the tests used in the assessments, it is possible to suggest that the risk of falls has been decreased after ten sessions of treadmill walking training, twice a week.

Gait speed is an indicator of general health status [38] and a strong predictor of risk for developing dementia [39] and falls [40] among older adults. Improvement in gait speed has been associated with longer survival in older adults [41]. A significant improvement of 0.21 m/s (24.4%) in gait speed indicated that our exercise program was efficient in improving balance performance. This improvement is larger than the meaningful change in gait speed (0.04 m/s) among older adults reported in other studies [42, 43]. In a study with frailty older adults submitted to a treadmill walking training, Oh-Park et al. found an increase of 18.8% in gait speed compared with pre- and posttreatment. Improvement in SPPB, BBS, and TUG scores demonstrated that the training increased the postural balance [44].

Some factors may explain the improved patterns of postural balance with treadmill training. One of them is that treadmill training has the ability to promote motor relearning and consequently to improve locomotor capacity during gait [45]. It has also been suggested that training with the repetitive movements generated by the treadmill activates locomotor patterns of functional movements, sensory inputs, and circuits of the central nervous system [46]. In addition, it has been hypothesized that repetitive movements associated with cutaneous and proprioceptive impulses may activate the generation of central movement patterns and in the long term, potentiate the motor cortex, facilitating motor learning [47].

The use of a treadmill (with or without partial weight support) permits a greater number of phases to be held in a training session, increasing the quantity of specific tasks [48]. For example, Hesse & Werner reported that patients who are victims of stroke performed up to 1000 steps in a 20-minute session of treadmill training compared with 50 to 100 steps during a 20-minute session of conventional physiotherapy [49]. In addition, the treadmill speed can be adjusted in order to reach a sufficient training intensity according to the capacity of each patient.

The effect of treadmill walking training alone on gait and balance parameters in institutionalized elderly people has not been described in the literature. This study shows that it is possible to include treadmill training in these patients, in addition to physiotherapeutic treatment to improve postural balance.

## 5. Conclusions

The present results permit us to conclude that a treadmill walking program had a positive effect on the postural balance of institutionalized older adults.

## Figures and Tables

**Table 1:** Baseline demographic data of the participants and group comparisons (mean and SD).

Variables	Intervention group (*n*=23)	Control group (*n*=14)	*p*
Sex			
Female	10 (43.5%)	7 (50%)	
Male	13 (56.5%)	7 (50%)	
Age (years)			
Females	77.8 ± 6.59	82.57 ± 10.50	0.26
Males	74.15 ± 8.56	74.00 ± 8.06	0.96
Weight (kg)	71.63 ± 15.20	66.98 ± 13.05	0.35
Height (m)	1.62 ± 0.11	1.60 ± 0.11	0.57
Number of medications	5.65 ± 3.69	7.07 ± 4.68	0.31
Duration of institutionalization (months)	32.74 ± 35.18	35.57 ± 28.94	0.56
MoCa score	26.61 ± 1.09	26.71 ± 1.43	0.93

SD: standard deviation; MoCa: montreal cognitive assessment.

**Table 2:** Results of the short physical performance battery, Berg balance scale, timed up and go, and gait speed test obtained for the intervention group^a^.

	Intervention group		
	Pretraining	Posttraining	1 month of follow-up
*SPPB*	8.26 ± 2.91	10.3 ± 2.20^*∗*^	10.43 ± 2.17^*∗*^
*BBS*	48.87 ± 8.49	52.13 ± 5.28^*∗*^	51.91 ± 5.58^*∗*^
*TUG*	14.04 ± 6.36	11.36 ± 4.73^*∗*^	11.65 ± 5.09^*∗*^
*GS*	0.86 ± 0.27	1.07 ± 0.30^*∗*^	1.03 ± 0.29^*∗*^

SPPB: short physical performance battery; BBS: Berg balance scale; TUG: timed up and go; GS: gait speed; ^*∗*^*p* < 0.001 compared with pretraining;^a^one-way repeated measures ANCOVA adjusted for age and baseline values as covariates.

**Table 3:** Comparison of the variables before and after intervention for the intervention group and the control group.

	Pretraining			Posttraining		
	Intervention group	Control group	*p*	Intervention group	Control group	*p*
SPPB	8.26 ± 2.91	6.28 ± 2.61	0.04	10.30 ± 2.20	7.71 ± 2.20	<0.001
BBS	48.87 ± 8.49^a^	46.50 ± 7.24^a^	0.770	51.51 ± 0.57^a^	46.79 ± 0.74^a^	<0.001
TUG (s)	14.05 ± 6.36^a^	15.45 ± 7.01^a^	0.540	11.79 ± 4.73^a^	15.71 ± 6.76^a^	<0.001
GS (m/s)	0.86 ± 0.27^a^	0.78 ± 0.32^a^	0.981	1.04 ± 0.30^a^	0.85 ± 0.29^a^	<0.001

SPPB: short physical performance battery; BBS: Berg balance scale; TUG: timed up and go; GS: gait speed. ^a^One-way ANCOVA adjusted for age and baseline values as covariates.

## Data Availability

All data used to support the findings of this study are included within the article.

## Additional Points

*Study limitations*. The study provides some limitations: first, the nonrandomization of participants due to ethical issues; second, the different number of participants in the intervention and control group; third, a brief follow-up period (1 month); and fourth, results cannot be generalized to all nursing home residents due to eligibility criteria (good cognitive performance of participants).

## References

[1] Abreu R. S., Naves E. L. M., Caparelli T. B., Mariano D. T. G., Dionísio V. C. (2014). Is it possible to identify the gender and age group of adults from gait analysis with hip-knee cyclograms?. *Revista Brasileira de Engenharia Biomédica*.

[2] Abreu R. S. (2013). *Análise Cinemática da Marcha em Esteira por meio de Ciclogramas em Jovens e Idosos de Ambos os Gêneros*.

[3] Silva J. R. d., Camargo R. C. T., Nunes M. M., Faria C. R. S. d., Abreu L. C. d. (2014). Análise da alteração do equilíbrio, da marcha e o risco de queda em idosos participantes de um programa de fisioterapia. *Colloquium Vitae*.

[4] Dorfman M., Herman T., Brozgol M. (2014). Dual-task training on a treadmill to improve gait and cognitive function in elderly idiopathic fallers. *Journal of Neurologic Physical Therapy*.

[5] Sarmento W., Sobreira F., Oliveira A. (2014). Avaliação do equilíbrio e da mobilidade de idosos após um programa de escola de posturas. *Revista Brasileira de Ciências da Saúde*.

[6] Silva H. E. d., Zipperer A. (2013). A correlação entre o desempenho físico funcional de membros inferiores e a gravidade da doença pulmonar obstrutiva crônica. *Fisioterapia em Movimento*.

[7] Novaes R. D., Miranda A. S., Dourado V. Z. (2011). Usual gait speed assessment in middle-aged and elderly Brazilian subjects. *Revista Brasileira de Fisioterapia*.

[8] da Silva Borges E. G., de Souza Vale R. G., Cader S. A. (2014). Postural balance and falls in elderly nursing home residents enrolled in a ballroom dancing program. *Archives of Gerontology and Geriatrics*.

[9] Faber L. M. (2017). *Comparação do Equilíbrio Estático e Mobilidade entre Idosos Institucionalizados e Idosos Moradores da Comunidade*.

[10] Pereira C., Baptista F., Cruz-Ferreira A. (2016). Role of physical activity, physical fitness, and chronic health conditions on the physical independence of community-dwelling older adults over a 5-year period. *Archives of Gerontology and Geriatrics*.

[11] Valenzuela T. (2012). Efficacy of progressive resistance training interventions in older adults in nursing homes: a systematic review. *Journal of the American Medical Directors Association*.

[12] Weening-Dijksterhuis E., de Greef M. H. G., Scherder E. J. A., Slaets J. P. J., van der Schans C. P. (2011). Frail institutionalized older persons: a comprehensive review on physical exercise, physical fitness, activities of daily living, and quality-of-life. *American Journal of Physical Medicine & Rehabilitation*.

[13] de Souto Barreto P., Morley J. E., Chodzko-Zajko W. (2016). Recommendations on physical activity and exercise for older adults living in long-term care facilities: a taskforce report. *Journal of the American Medical Directors Association*.

[14] Morley J. E., Caplan G., Cesari M. (2014). International survey of nursing home research priorities. *Journal of the American Medical Directors Association*.

[15] Ganesan M., Sathyaprabha T. N., Pal P. K., Gupta A. (2015). Partial body weight-supported treadmill training in patients with Parkinson disease: impact on gait and clinical manifestation. *Archives of Physical Medicine and Rehabilitation*.

[16] Lindquist A. R., Prado C. L., Barros R. M., Mattioli R., da Costa P. H. L., Salvini T. F. (2007). Gait training combining partial body-weight support, a treadmill, and functional electrical stimulation: effects on poststroke gait. *Physical Therapy*.

[17] Sales V. (2014). *Avaliação do Efeito do Treino de Marcha em Esteira com e sem Suspensão do Peso Corporal no Equilíbrio de Pacientes com Doença de Parkinson em Uso de Estimulação Cerebral Profunda*.

[18] van Ooijen M. W., Roerdink M., Trekop M., Visschedijk J., Janssen T. W., Beek P. J. (2013). Functional gait rehabilitation in elderly people following a fall-related hip fracture using a treadmill with visual context: design of a randomized controlled trial. *BMC Geriatrics*.

[19] Hesse S. (2008). Treadmill training with partial body weight support after stroke: a review. *Neurorehabilitation*.

[20] Schindl M. R., Forstner C., Kern H., Hesse S. (2000). Treadmill training with partial body weight support in nonambulatory patients with cerebral palsy. *Archives of Physical Medicine and Rehabilitation*.

[21] Sauvage L. R., Myklebust B. M., Crow-Pan J. (1992). A clinical trial of strengthening and aerobic exercise to improve gait and balance in elderly male nursing home residents. *American Journal of Physical Medicine & Rehabilitation*.

[22] Nowalk M. P., Prendergast J. M., Bayles C. M., D’Amico F. J., Colvin G. C. (2001). A randomized trial of exercise programs among older individuals living in two long-term care facilities: the fallsFREE program. *Journal of the American Geriatrics Society*.

[23] Tsaih P.-L., Shih Y.-L., Hu M.-H. (2012). Low-intensity task-oriented exercise for ambulation-challenged residents in long-term care facilities: a randomized, controlled trial. *American Journal of Physical Medicine & Rehabilitation*.

[24] Izquierdo M., Rodriguez-Mañas L., Casas-Herrero A., Martinez-Velilla N., Cadore E. L., Sinclair A. J. (2016). Is it ethical not to precribe physical activity for the elderly frail?. *Journal of the American Medical Directors Association*.

[25] Holden M. K., Gill K. M., Magliozzi M. R., Nathan J., Piehl-Baker L. (1984). Clinical gait assessment in the neurologically impaired: reliability and meaningfulness. *Physical Therapy*.

[26] Cecato J. F., Montiel J. M., Bartholomeu D., Martinelli J. E. (2014). Poder preditivo do MoCa na avaliação neuropsicológica de pacientes com diagnóstico de demência. *Revista Brasileira de Geriatria e Gerontologia*.

[27] Nasreddine Z. S., Phillips N. A., BÃ©dirian V. r. (2005). The montreal cognitive assessment, MoCA: a brief screening tool for mild cognitive impairment. *Journal of the American Geriatrics Society*.

[28] Podsiadlo D., Richardson S. (1991). The timed “Up and Go” test: a test of basic functional mobility for frail elderly persons. *Journal of the American Geriatrics Society*.

[29] Schoene D., Wu S. M.-S., Mikolaizak A. S. (2013). Discriminative ability and predictive validity of the timed up and go test in identifying older people who fall: systematic review and meta-analysis. *Journal of the American Geriatrics Society*.

[30] Miyamoto S. T., Lombardi Junior I., Berg K. O., Ramos L. R., Natour J. (2004). Brazilian version of the Berg balance scale. *Brazilian Journal of Medical and Biological Research*.

[31] Nakano M. M. (2007). *Versão Brasileira da Short Physical Performance Battery– SPPB: Adaptação Cultural e Estudo da Confiabilidade*.

[32] Bello O., Sanchez J. A., Fernandez-del-Olmo M. (2008). Treadmill walking in Parkinson’s disease patients: adaptation and generalization effect. *Movement Disorders*.

[33] Ikezoe T., Asakawa Y., Shima H., Kishibuchi K., Ichihashi N. (2013). Daytime physical activity patterns and physical fitness in institutionalized elderly women: an exploratory study. *Archives of Gerontology and Geriatrics*.

[34] Bulat T., Hart-Hughes S., Ahmed S. (2007). Effect of a group-based exercise program on balance in elderly. *Clinical Interventions in Aging*.

[35] Judge J. (2003). Balance training to maintain mobility and prevent disability. *American Journal of Preventive Medicine*.

[36] Karlsson M., Nordqvist A., Karlsson C. (2008). Physical activity, muscle function, falls and fractures. *Food & Nutrition Research*.

[37] Sherrington C., Michaleff Z. A., Fairhall N. (2017). Exercise to prevent falls in older adults: an updated systematic review and meta-analysis. *British Journal of Sports Medicine*.

[38] Studenski S., Perera S., Patel K. (2011). Gait speed and survival in older adults. *JAMA*.

[39] Verghese J., Lipton R. B., Hall C. B., Kuslansky G., Katz M. J., Buschke H. (2002). Abnormality of gait as a predictor of Non-Alzheimer’s dementia. *New England Journal of Medicine*.

[40] Verghese J., Holtzer R., Lipton R. B., Wang C. (2009). Quantitative gait markers and incident fall risk in older adults. *The Journals of Gerontology Series A: Biological Sciences and Medical Sciences*.

[41] Hardy S. E., Perera S., Roumani Y. F., Chandler J. M., Studenski S. A. (2007). Improvement in usual gait speed predicts better survival in older adults. *Journal of the American Geriatrics Society*.

[42] Brach J. S., Perera S., Studenski S., Katz M., Hall C., Verghese J. (2010). Meaningful change in measures of gait variability in older adults. *Gait & Posture*.

[43] Perera S., Mody S. H., Woodman R. C., Studenski S. A. (2006). Meaningful change and responsiveness in common physical performance measures in older adults. *Journal of the American Geriatrics Society*.

[44] Oh-Park M., Holtzer R., Mahoney J., Wang C., Verghese J. (2011). Effect of treadmill training on specific gait parameters in older adults with frailty: case series. *Journal of Geriatric Physical Therapy*.

[45] Visintin M., Barbeau H. (1989). The effects of body weight support on the locomotor pattern of spastic paretic patients. *Canadian Journal of Neurological Sciences/Journal Canadien des Sciences Neurologiques*.

[46] Dietz V. (2003). Spinal cord pattern generators for locomotion. *Clinical Neurophysiology*.

[47] Asanuma H., Keller A. (1991). Neuronal mechanisms of motor learning in mammals. *Neuroreport*.

[48] Mehrholz J., Thomas S., Elsner B. (2017). Treadmill training and body weight support for walking after stroke. *Cochrane Database of Systematic Reviews*.

[49] Hesse S., Werner C. (2003). Poststroke motor dysfunction and spasticity: novel pharmacological and physical treatment strategies. *CNS Drugs*.

